# Conservative Treatment or Surgical Treatment: A Case Report and Literature Review of Multiple Fractures of the Lower Extremities in a Child with Insensitivity to Pain

**DOI:** 10.1111/os.12676

**Published:** 2020-04-19

**Authors:** Yi‐kang Yu, Dong‐peng Tu, Xiao‐lin Shi, Zheng Liu, Xin Fan, Chao Xu

**Affiliations:** ^1^ Zhejiang Chinese Medical University, Second Clinical Medical College of Zhejiang Chinese Medical University Zhejiang China; ^2^ Department of Orthopaedics Xinhua Hospital of Zhejiang Province, The Second Affiliated Hospital of Zhejiang Chinese Medical University Zhejiang China

**Keywords:** Comprehensive treatment, Congenital pain insensitive, Fracture, Hereditary sensory autonomic neuropathy, NTKRI gene

## Abstract

Congenital pain insensitivity is a rare genetic disease and its clinical manifestations are many. In orthopaedics, common complications of this disease include painless fracture and Charcot's arthropathy. We followed up a case of multiple fractures of the lower extremity in two years, during which time he came to the clinic for five painless fractures of the lower extremity in a total of six parts. A mutation was found on the NTKRI gene (chr1:156813923(hg19), NM_001007792.1: c.1221938C > T). We have developed a combination of surgery and conservative treatments for his condition, focusing on the mental state of the child and considering comprehensive treatment to be the best option for this type of patient. Occult fractures caused by pain insensitivity are often treated only as fractures, however their complications require routine examination and cleaning, suitable protective shoes, splint fixation, stretching, guided exercise planning, and early treatment of injuries. Due to the risk of fracture in the future, it is important that parents pay attention to the behavior and psychology of the child, such as not letting the child participate in exercise with a risk of injury, protective measures while playing, engaging in psychological counseling, and inducing interest in mental activity. These interventions will play a very important role in preventing the recurrence of fracture.

## Introduction

Congenital pain insensitivity (CIP) is a rare nervous system disease characterized by varying degrees of sensory loss and autonomic nervous dysfunction. This situation was first described by Dearborn in 1932^1^. The main cause of CIP is hereditary sensory autonomic neuropathy (HSAN) which was named by Dyck. And he divided it into five different types, and the latest classification increased it to eight types^2^, ^3^. The clinical findings of our case fit the HSAN‐IV type, the clinical manifestations are insensitivity to pain, self‐harm behavior, mental impairment, no sweat, hyperactivity and emotional changes^4^, ^5^. In this case, the clinical manifestations include recurrent lower limb fractures, insensitive to the fracture pain, biting fingers, and unstable mood. Similar reports suggest that some CIP patients may also recover within a period of time after pain loss, and the degree of sensory loss varies^6^.

## Case Report

**Figure 1:**
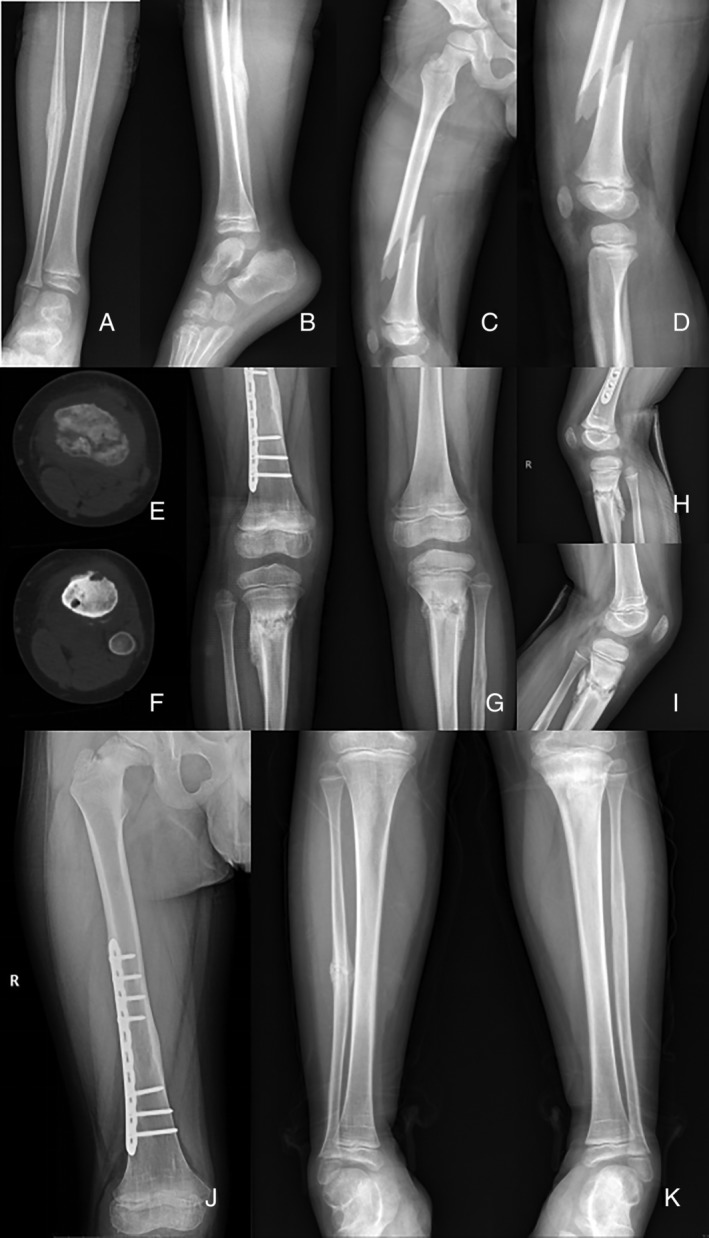
(A,B) Old fracture of the middle part of the right fibula, eschar formation (X‐ray positive and lateral position). (C,D) Right middle and lower thigh fracture, spiral fracture line (X‐ray positive and lateral position). (E,F) Bilateral proximal tibia fractures, CT showed cavity formation in the bone marrow cavity. (G,H,I) Bilateral proximal tibia fractures, the fracture site showed a large number of eschar formation (X‐ray positive and lateral position). (J) The old fracture of the right thigh neck, the shortening of the neck of the thigh. (K) Old fracture of the middle part of the fibula on the right, scab formation.

We have a special case of a multiple‐fracture patient, male, born on 26 October 2011, Chinese, Han nationality. The child had a history of repeated and multiple lower extremity fractures which started at 5 years old (five times in 2 years, including six fractures in six parts). These fractures were usually accompanied with emotional instability, impatience and hyperactivity, biting fingers, and so on. According to the order of fracture, the fractures were in the middle part of the right fibula, the middle and lower segment of the right thigh, the bilateral upper tibia, the right neck of the thigh, and the middle part of the right fibula (Fig. 1, Table 1). There can be an obvious history of trauma or no history of trauma at the time of fracture. The fractures are mostly mild violent damage, and there may be temporary pain after injury.

**Table 1:** History of multiple fractures in child

Age	Fracture site	Cause	Performance and process	Treatment
Five years and 4 months	The middle part of the fibula on the right (Fig. 1A,B)	To fall; a slight violence	One month after the fall, an abnormal posture was found in the right calf walking, during which there was no obvious pain, and there was an old fracture at the time of the visit.	Conservative treatment; plaster external fixation.
Six years and 1 months	Right femoral lower section (Fig. 1C,D)	Drop from a height of about 1 m	Pain, deformity, limp when falling, see a doctor in 2 h	Open reduction and internal fixation; Use a plate as implantation; large number of hyperplastic eschar during operation
Six years and 8 months	Bilateral proximal tibia (Fig. 1E–I)	To fall; a slight violence	After falling, there was no limp and the pain was not obvious. CT showed cavity in bone marrow cavity	Manual reduction, Conservative treatment, plaster external fixation
Six years and 11 months	Right thigh neck (Fig. 1J)	Accidental discovery in review	No obvious trauma, pain is not obvious, X‐ray examination showed old fracture	Manual reduction, Conservative treatment, manual reduction 1–2 weeks (reexamination for 6 months), plaster external fixation
7 years and 6 months	The middle part of the fibula on the right (Fig. 1K)	Accidental discovery in review	No obvious trauma, pain is not obvious, X‐ray examination showed old fracture	Conservative treatment, plaster external fixation

**Figure 2:**
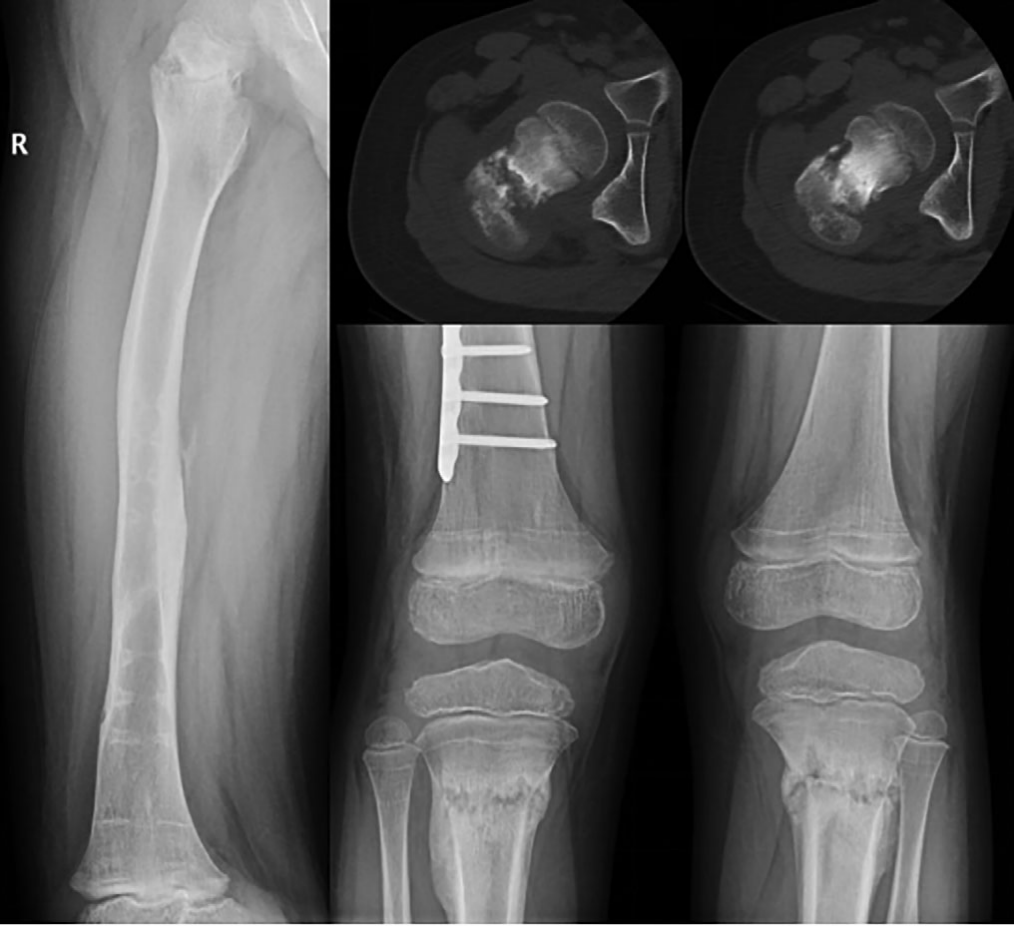
Twelve weeks after the right femoral neck fracture was found, the positive lateral position of the X‐ray showed the formation of the femoral neck of the femoral neck; the CT showed the cystic change of the femoral neck; 4 weeks after the two‐sided proximal fracture of the tibia, the X‐ray showed that the growth of the positive‐position bone was higher than before.

During physical examination, the tenderness pain at the fracture site was not obvious, the longitudinal percussion pain at the fracture site was also not obvious, and, most of the time, the child could walk by himself with the multiple fractures of lower limbs. Among them, the fracture of the right thigh neck and the fracture of the middle part of the right fibula are fractures caused by unknown causes. They were found to be in a healing state at the time of discovery. When the original fracture was reexamined, it was found that the pain was not obvious. The process of fracture healing showed fast healing and rapid growth of eschar, and the cystic change can be founded in Fig. 2.

Laboratory and imaging examination showed that bone mineral density, bone metabolism, 25‐hydroxyvitamin D, serum electrolytes (calcium, phosphorus, magnesium), and serum parathyroid were all within the normal range. After sequencing the whole gene of the child and his parents, filtering out the high frequency variation in the population, and checking the data of the child's family, it was found that there was a heterozygous variation in the depth of the intron of the NTRK1 gene:chr1:156813923(hg19), NM_001007792.1:c.122+1938C>T. The mother was also a heterozygous carrier, but did not show the phenotype of the related disease.

We took internal fixation to treat the right thigh shaft fracture, and removed the internal fixation after healing, but during this period, we found the fractures of bilateral tibia and right thigh neck, so the subsequent fractures were treated conservatively by manual reduction and external fixation. After a fracture of the right thigh neck was found in the child, we adopted manual reduction treatment and outpatient follow‐up. We used regular weekly X‐ray reexamination, the manipulative reduction of the lower extremities of the child according to the examination results, we reduced fracture by hand, as well as the adjusted of external fixations.

**Figure 3:**
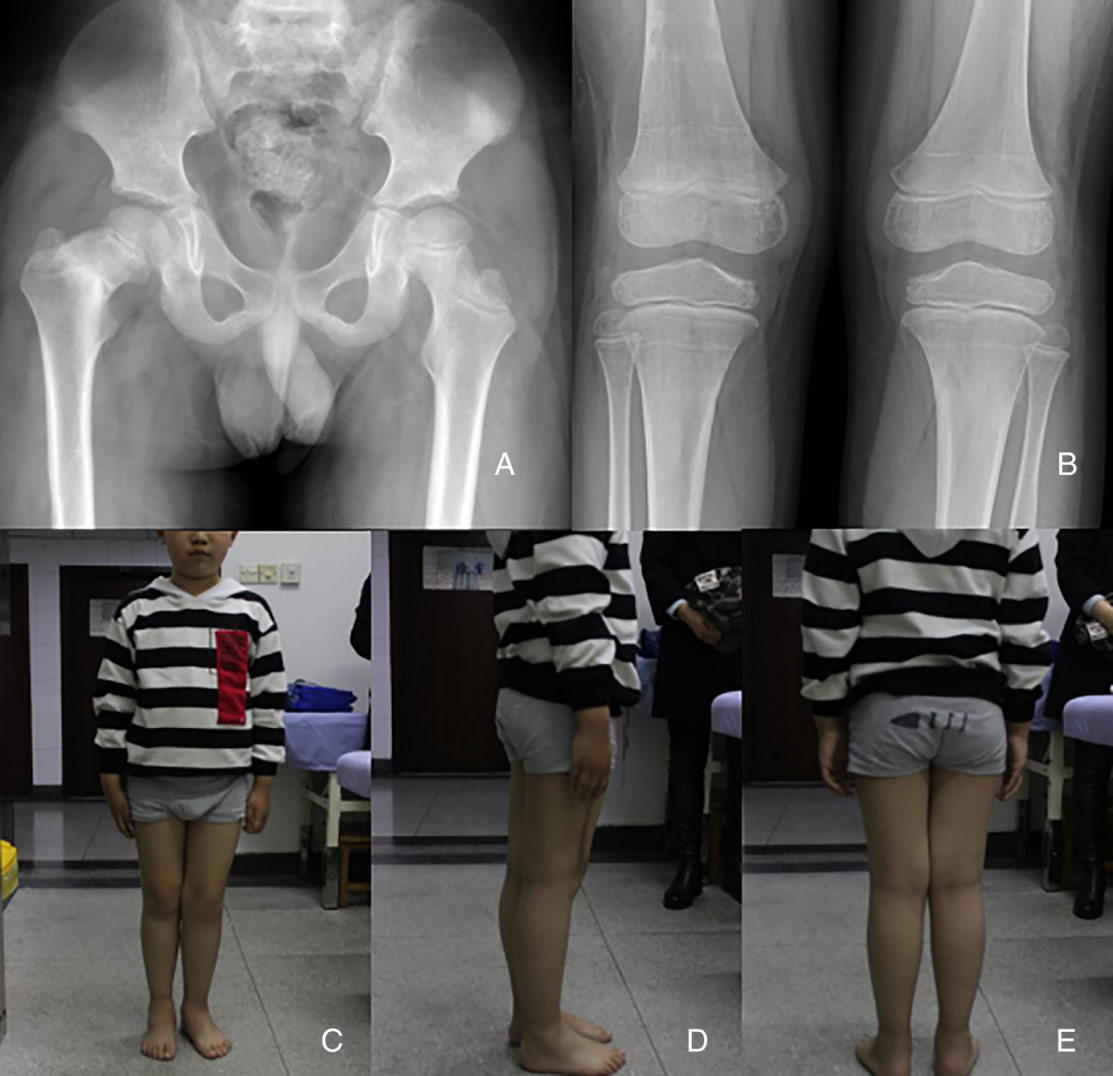
(A) Thirty‐two weeks after conservative treatment, the right thigh neck shortening and internal rotation were found. (B) Twenty‐four weeks after bilateral proximal tibia fracture was found and conservative treatment, the right thigh neck fracture was continuous. (C,D,E) At the bilateral fracture site for 26 weeks. After 38 weeks of bilateral tibia fracture, the internal circumflex muscle of the right lower extremity atrophied slightly, and the shortening of the right lower extremity was not obvious.

After treatment, the deformity of the right lower extremity was basically corrected, and the shortening of the right lower extremity was not obvious (Fig. 3). After removing the external fixation, we examined the child in detail again: the child was 7 years and 5 months old, the body length was 134.1 cm, the weight was 36 kg, the total length of the right lower extremity was 69 cm, the length of the left lower extremity was 70 cm, there was no obvious tenderness pain, no obvious swelling, and right lower limb was slight varus. The trunk angle of the right thigh neck is about 122°. Other evaluations and results include percussion pain of the longitudinal axis of both lower extremities (−), pap sign (−), Patrick sign (+) of right lower extremity, normal muscle strength of the right lower extremity, no obvious abnormality in nervous system examination, temperature sense of extremities, touch sense, and superficial skin pain feeling. After fracture healing, the child had good exercise ability.

## Discussion

The most common mutation of the HSAN‐IV type in the Chinese Han population is caused by the NTRK 1 gene mutation, and the most common mutation is c.851‐33T> A, which accounts for 63% of the known population, and the incidence in the population carrying the mutation is concentrated in southern China^7^. We conducted a full exome sequencing of this patient and found that the c.122 + 1938 C> T heterozygous mutation in the patient was not described in the literature and that the child's mother was the carrier of the site mutation. Kurth *et al*. reported that a new splicing site was produced by C > A transversion caused by intron mutation, which led to the incorporation of 10 intron bases into NTRK1 mRNA and which bound to non‐functional gene products^8^. We believe that the cause of this patient is similar, due to heterozygous mutations in the intron, resulting in reduced protein expression, and the phenotype that produces sensory dysfunction. However, due to funding constraints, we did not extract the gene from this child and structurally analyze the gene expression product.

For this kind of children, symptomatic treatment has always been the first choice, as gene therapy treatments have not been developed^3^. Occult fractures caused by pain insensitivity are often treated only as fractures, but their complications require important considerations, such as routine examination and cleaning, suitable protective shoes, splint fixation, stretching, guided exercise planning, and early treatment of injuries^9^.

Because of the growth characteristics of children, the limb long bone force line correction and joint function recovery are difficult for surgeons to overcome in the surgical treatment of fractures. Small fractures caused by insensitivity to pain, such as in the case of Shackor's disease, in conjunction with fractures of the ankle, often involving epiphyseal injury, take the using of percutaneous minimally invasive internal fixation in order to avoid damage to the blood flow and original tissue structure of the site in children^10^, ^11^. Because of its load‐bearing function, early anatomical reduction and restoration of the original length are of great significance to the growth and development of children's bones. Current techniques such as open reduction combined with external fixator or endomedulary Titanium Elastic Nails (TEN)^12^, ^13^ are available to better restore the limb force line.

Due to the uncertainty of the occurrence time of the fracture event and the concealment of the fracture site, diagnosis of fractures become difficult. In this case, when the fracture of the middle and lower segment of the right thigh was found, the child was treated surgically. Due to the limitation of the material at that time, the elastic Kirschner wire could not be used to fix the fracture. However, the right femoral neck fracture found in the later period, and several subsequent fractures, were in the healing state at the time of discovery; also, the right femoral neck fracture could not exclude stress fracture after plate implantation, so we adopted the manual reduction and external fixation treatment. Delniotis *et al*. followed up a case of asymptomatic Charcot hip fracture caused by CIP syndrome for 10 years. It was mentioned that no matter what standard surgical treatment was used, the treatment of advanced hip dysplasia in CIP syndrome seemed ineffective^14^.

After examining the literature, we found that the best advice for fracture or dislocation of hip joint in patients with CIP syndrome is conservative treatment without surgical intervention, and early gypsum fixation can prevent or at least slow down the development of destructive hip dysplasia^15^, ^16^. However, in the case of non‐union of the bone in the CIP syndrome, the non‐cement‐type total hip replacement may be the best solution due to the non‐painful wear of the acetabulum in most patients following the hemiarthroplasty^17^.

Generally speaking, the sensory loss of the child is partial loss, which leads to the lack of obvious pain at the time of fracture, and it is easy to delay the discovery of fracture. The heterozygous mutation of NTRK 1 gene in c.122 1938C > T needs to be discussed. The surgical treatment we took made the spiral fracture of the right femoral shaft fixed, but the right femoral neck fracture, because of the presence of internal plants, means that manual reduction combined with external fixation is the best way to treat the child, as the treatment of child's right lower limb can basically restore the original function. Because of the future risk of fracture, parents must pay attention to the behavior and psychology of their children.These measures include preventing children from taking injury‐risk exercises, taking protective measures while playing, paying attention to their mental health, and making children interested in mental activities. These interventions will play a very important role in preventing the recurrence of fracture.
